# A randomized, open-label, two-period crossover study to evaluate the bioequivalence and food effect between two formulations of regorafenib in healthy adult participants

**DOI:** 10.3389/fphar.2025.1511558

**Published:** 2025-03-19

**Authors:** Yan Li, Lu Qi, Caixia Yang, Na Zhao, Xinghe Wang

**Affiliations:** ^1^ Phase I Clinical Trial Center, Beijing Shijitan Hospital, Capital Medical University, Beijing, China; ^2^ Beijing SL Pharmaceutical Co., Ltd., Beijing, China

**Keywords:** bioequivalence, food effect, pharmacokinetics, healthy subjects, regorafenib

## Abstract

**Background:**

This research aimed to compare the bioequivalence of a test formulation (regorafenib produced by Beijing SL Pharmaceutical Co., Ltd.) with a reference formulation (the original drug Stivarga^®^) in Chinese healthy subjects under fasting conditions and two postprandial states: after low-fat and high-fat meals.

**Methods:**

The research design was a randomized, open-label, two-period crossover trial involving a single 40 mg oral dose. Three separate studies were conducted. Study 1 enrolled 64 subjects who were dosed under fasting conditions; Study 2 involved 76 subjects dosed after a low-fat breakfast; and Study 3 also involved 76 subjects dosed after a high-fat breakfast. Plasma concentrations of regorafenib and M-2 were determined using a liquid chromatography-tandem mass spectrometry (LC-MS/MS) method. The primary endpoints were the peak plasma concentration (C_max_), the area under the concentration-time curve from time 0 to 168 h (AUC_0–168h_), and the extrapolated area under the curve from time zero to infinity (AUC_0–∞_) of regorafenib, with pharmacokinetics (PK) parameters of the metabolite M-2 serving as reference data.

**Results:**

The results showed that, under fasting, post-low-fat meal, and post-high-fat meal conditions, the 90% confidence intervals (CIs) of geometric mean ratios (GMRs) for C_max_ of test to reference regorafenib were 96.39%–114.94%, 93.81%–106.67% and 94.23%–107.21%, respectively. For AUC_0–168h_ were 88.40%–102.04%, 92.40%–102.97% and 92.50%–102.60%. For AUC_0–∞_ were 85.86%–100.01%, 90.26%–101.79% and 90.15%–101.36%. All of these fell within the 80.00%–125.00% range, meeting the equivalence criteria. Food intake had some impact on the PK parameters of regorafenib, but the effect was minor. Administration of a single 40 mg dose of regorafenib to healthy subjects demonstrated good safety and tolerability.

**Conclusion:**

Under different dietary conditions, a single oral dose of 40 mg of generic drug regorafenib was bioequivalent to the original drug Stivarga^®^ in healthy Chinese subjects, and the food effect was limited.

**Clinical Trial Registration:**

http://www.chinadrugtrials.org.cn/, identifier CTR20210575, CTR20210576, CTR20223278.

## Introduction

Regorafenib (Stivarga^®^) is an orally administered, multi-targeted tyrosine kinase inhibitor (TKI) capable of inhibiting angiogenesis and other oncogenic pathways, including vascular endothelial growth factor receptors 1–3 (VEGFR1-3), platelet-derived growth factor receptors (PDGFR), fibroblast growth factor receptors (FGFR), the angiopoietin receptor TIE-2, and additional tyrosine kinase receptors and proteins (Strumberg and Schultheis, 2012). By blocking these pathways, regorafenib disrupts tumor angiogenesis, curbs tumor cell proliferation, and modulates the tumor microenvironment, thereby hindering tumor growth and invasion. Its clinical efficacy has been demonstrated in several studies, notably the CORRECT trial (Grothey et al., 2013) for metastatic colorectal cancer (mCRC), the RESORCE trial (Bruix et al., 2017) for hepatocellular carcinoma (HCC), and the GRID trial (Demetri et al., 2013) for gastrointestinal stromal tumors (GIST). Consequently, the U.S. Food and Drug Administration (FDA) granted approvals for these indications between 2012 and 2017.

Pharmacokinetic (PK) studies have established that regorafenib exhibits specific characteristics following administration. After a single 160 mg dose of Stivarga^®^ in patients with advanced solid tumors, regorafenib achieves a geometric mean peak plasma concentration (C_max_) of 2.5 μg/mL within approximately 4 hours, alongside a geometric mean area under the plasma concentration-time curve (AUC) of 70.4 μg h/mL (Stivarga^®^ (regorafenib) Accessdata, 2012; Stivarga^®^ summary. 2013). Cytochrome P450 3A4 (CYP3A4) and uridine diphosphate glucuronosyltransferase 1A9 (UGT1A9) are involved in the metabolism of regorafenib. The primary circulating metabolites found in human plasma are M-2 (N-oxide) and M-5 (N-oxide and N-desmethyl), both possessing *in vitro* pharmacological activity (Zopf et al., 2016). Moreover, because of enterohepatic circulation, the metabolites may be reduced or hydrolyzed by microorganisms in the gastrointestinal tract, and the main products of this process are repafenib and M-2. The pharmacological activity of M-2 is similar to that of the prototype of regorafenib, and stronger than that of M-5. M-2 contributes significantly to the total systemic exposure, accounting for approximately 30%–40% of the parent drug’s AUC, while M-5 contributes less than 10%.

Food intake may significantly impacts the pharmacokinetics of regorafenib. A study [Stivarga^®^ (regorafenib) Accessdata, 2012] involving 24 healthy males demonstrated that consuming a high-fat meal along with a single 160 mg dose of Stivarga^®^ led to a 48% increase in the mean AUC of regorafenib, while decreasing the mean AUC of M-2 and M-5 metabolites by 20% and 51%, respectively, compared to the fasted state.

Developed by Bayer AG, Stivarga^®^ received FDA approval in 2012 and is currently marketed in approximately one hundred countries. Given the significant patient populations with mCRC, HCC, and GIST in China (Chen et al., 2016), regorafenib gained approval for use in China in 2017. Despite its efficacy, the high cost of the branded medication has limited accessibility for many patients. With the expiration of the regorafenib patent in 2024, efforts are underway to evaluate its PK profile in the Chinese population to support the introduction of a generic version. This research included administering regorafenib under three conditions: fasting, post low-fat meal, and post high-fat meal, reflecting the drug’s recommended administration with a low-fat meal as per the Stivarga^®^ prescribing information.

## Methods

### Ethics

The research was conducted at Beijing Shijitan Hospital, Beijing, China. The study protocols and informed consent forms were reviewed and approved by the Institutional Review Board (IRB) of Beijing Shijitan Hospital (2021(6), 2021(7), 2022(41)). The research adhered to ethical principles derived from international guidelines, including the Declaration of Helsinki and the International Council for Harmonisation Guidelines for Good Clinical Practice (ICH GCP), as well as all applicable legal and regulatory requirements. All participants provided written informed consent prior to the initiation of any procedures. The studies were registered at chinadrugtrials. org.cn (Study Numbers: CTR20210575, CTR20210576, CTR20223278; date: 25 March 2021, 26 March 2021, 27 December 2022).

### Formulations

The test formulation used was a regorafenib tablet manufactured by Beijing SL Pharmaceutical Co., Ltd., Beijing, China (40 mg/tablet, batch number: 20200102, expiry date: 9 July 2023). The reference formulation was Stivarga^®^ from Bayer AG (40 mg/tablet; for studies 1 and 2, batch number: BXJAZ32, expiry date: 13 August 2022; for study 3, batch number: BXJRNL4, expiry date: 5 October 2024).

### Participants

Eligible participants were healthy Chinese males aged over 30 years with a Body Mass Index (BMI) within the range of 19.0–28.0 kg/m^2^ at screening. Participants were deemed healthy based on a comprehensive clinical assessment, which included a detailed medical history review, thorough physical examination, vital signs monitoring, electrocardiogram (ECG) analysis, and standard laboratory evaluations. Inclusion criteria also required participants to agree to use an effective method of contraception throughout the study period. Participants were prohibited from using concurrent medications, consuming alcohol, smoking tobacco, or taking dietary supplements during the studies. Individuals who had taken drugs known to induce or inhibit drug-metabolizing enzymes (such as barbiturates) within 30 days prior to the administration were excluded. Additionally, those with a history of liver disease or elevated levels of alanine aminotransferase (ALT) and aspartate aminotransferase (AST) above the upper limit of normal, or with a history of allergic disease were not included in the studies.

### Study design and treatment

Three separated studies involving 216 healthy Chinese male participants under varying dietary conditions (fasting, post low-fat breakfast, post high-fat breakfast) were conducted. Each study was a randomized, single-center, open-label, two-treatment, two-period, two-sequence crossover study with a 12-day washout interval. Given the half-lives of regorafenib and its metabolite M-2 (approximately 25 and 28 h, respectively) [Stivarga^®^ (regorafenib) label. 2012; Stivarga^®^ summary. 2013], the washout period was set at 12 days to ensure complete elimination of the drug prior to the next dosing period. According to the requirements of the Chinese National Medical Products Administration (NMPA), the dosage should be the maximum specification of a single tablet. Since the only specification for regorafenib tablets is 40mg, the single-dose in the studies was set at 40 mg. SAS statistical software (v9.4) generated a random number table assigning participants to either sequence A (Test (T)/Reference (R)) or B (R/T) in a 1:1 ratio. Sixty-four participants were randomized into Study 1 under fasting conditions, while 76 participants each were randomized into Studies 2 and 3 under low-fat and high-fat fed conditions, respectively. Subjects were allocated random numbers sequentially based on their screening numbers. Due to preclinical findings of increased necrotic corpora lutea in female rats’ ovaries, only male adults were enrolled.

In Study 1, all participants received the regorafenib tablet (40 mg) or Stivarga^®^ (40 mg) under fasting conditions (after at least a 10-h fast) on day 1 of each period. In Studies 2 and 3, participants received the regorafenib tablet or Stivarga^®^ under fed conditions. Specifically, Study 2 utilized a low-fat breakfast consisting of a boiled egg, a slice of bread, and 250 mL of skimmed milk (totaling 411 kcal with 25, 380, and 6 kcal from protein, carbohydrates, and fat, respectively). Study 3 involved a high-fat breakfast comprising an egg, a piece of beef cooked with oil, a steamed bun, and 250 mL of whole milk (totaling 925 kcal with 150, 265, and 510 kcal from protein, carbohydrates, and fat, respectively). Breakfasts were designed to align with FDA recommendations regarding calorie and fat content (FDA, 2021; Parsad and Ratain, 2017). Participants were required to complete their breakfast within 30 min and abstain from food for at least 4 h post-administration. Standardized meals were provided at least 4 and 10 h post-administration for lunch and dinner, respectively.

### Blood sample collection and PK analysis

Blood samples were collected at specified time points post-administration for all studies. In Study 1, venous blood (approximately 4 mL) was drawn at 18 predetermined intervals: 0 h (within 1 h pre-administration), 1 h, 2 h, 2.5 h, 3 h, 3.5 h, 4 h, 4.5 h, 5 h, 6 h, 8 h, 12 h, 24 h, 48 h, 72 h, 96 h, 120 h, and 168 h. The previous food effect study showed that T_max_ of regorafenib was delayed due to food effect, thus in Studies 2 and 3, a blood sampling points of 5.5 h was added and point two h was deleted. The blood samples were 0 h (within 1 h pre-administration), 1 h, 2.5 h, 3 h, 3.5 h, 4 h, 4.5 h, 5 h, 5.5 h, 6 h, 8 h, 12 h, 24 h, 48 h, 72 h, 96 h, 120 h, and 168 h.

Blood samples were centrifuged at 2°C–8°C and 1,700 g for 10 min. Plasma was separated and divided into two parts: one for testing and one for backup storage. Samples were stored at −20°C and later transferred to a long-term storage freezer at −60 to −90°C prior to pharmacokinetic analysis.

Plasma samples were analyzed at Wuhan Hongren Biopharmaceutical Inc. (Wuhan, China) using a validated liquid chromatography-tandem mass spectrometry (LC-MS/MS) method. Briefly, regorafenib and M-2 were quantified by mixing 50 μL of K_2_EDTA human plasma with 50 μL of internal standard followed by protein precipitation with acetonitrile. The supernatant was then transferred and diluted before injection into the LC-MS/MS system. Data were acquired and processed using Analyst 1.6.3 software, and sample management and regression calculations were performed using Watson^®^ LIMS software (version 7.5). The linearity range for regorafenib and M-2 was 3.00–1,500 ng/mL and 1.00–500 ng/mL, respectively. The interday precision (CV% coefficient of variation) for regorafenib was <5.4%, and the accuracy ranged within −2.3%–2.7%. The interday precision (CV% coefficient of variation) for M-2 was <6.8%, and the accuracy ranged within −2.0%–0.4%.

Primary pharmacokinetic parameters included the peak plasma concentration (C_max_), the area under the concentration-time curve from time zero to 168 h (AUC_0–168h_), and the extrapolated area under the curve from time zero to infinity (AUC_0–∞_) for regorafenib. Secondary parameters included time to reach C_max_ (T_max_), half-life (t_1/2_), elimination rate constant (λ_z_), and the same primary parameters for M-2. Due to the low blood concentration and pharmacological activity of M-5, the metabolite M-5 was not detected, only M-2 was measured.

### Safety assessment

Participants were continuously monitored through vital sign assessments, physical examinations, laboratory tests (including hematology, biochemistry, and urinalysis), and standard 12-lead electrocardiograms (ECGs). Adverse events (AEs) were graded using the Common Terminology Criteria for Adverse Events (CTCAE) version 5.0 and categorized by System Organ Class or Preferred Term according to the Medical Dictionary for Regulatory Activities (MedDRA).

### Statistical analysis

The sample size was determined based on statistical considerations. For Study 1, assuming the true ratio was 0.92 and the intra-individual coefficient of variation (Intra-CV) was 26% in a two-way crossover design, 52 participants were randomized to achieve at least 80% power for meeting the bioequivalence criteria. Considering a subject withdrawal rate of 20%, the number of participants was increased to 64. For Studies 2 and 3, assuming the true ratio was 0.9, Intra-CV was 28%, and other settings were consistent with those in the fasting study, the sample number was set at 76. Bioequivalence was assessed using a mixed-effects model with analysis of variance in SAS (version 9.4). Participants who had at least one evaluable pharmacokinetic parameter were included in the pharmacokinetic analysis set, while those who completed at least one treatment period were included in the bioequivalence analysis set. If there are cases where the extrapolated area percentage exceeds 20%, a sensitivity analysis should be conducted to further determine whether such data can be excluded. Primary endpoints (C_max_, AUC_0–168h_, and AUC_0–∞_ of regorafenib) were log-transformed, and the 90% confidence intervals (CIs) for the geometric mean ratios (GMRs) of the primary parameters were calculated. Bioequivalence was assumed if the 90% CIs of GMRs for C_max_, AUC_0–168h_, and AUC_0–∞_ of the prototype of regorafenib fell within the predefined range of 80.00%–125.00%. Descriptive statistics were provided for all other parameters.

## Results

### Participants

Study 1 was conducted from 22 September 2021, to 8 November 2021. Initially, sixty-four subjects were enrolled. However, two participants withdrew before the first administration due to personal reasons, and six discontinued participation due to adverse events (AEs) after the first period. Consequently, fifty-six subjects completed both treatment periods.

Study 2 ran from 16 June 2021, to 2 August 2021, with seventy-six subjects being enrolled. One participant withdrew before the first administration for personal reasons, and two subjects discontinued due to AEs. In total, seventy-three subjects completed both treatment periods.

**FIGURE 1:**
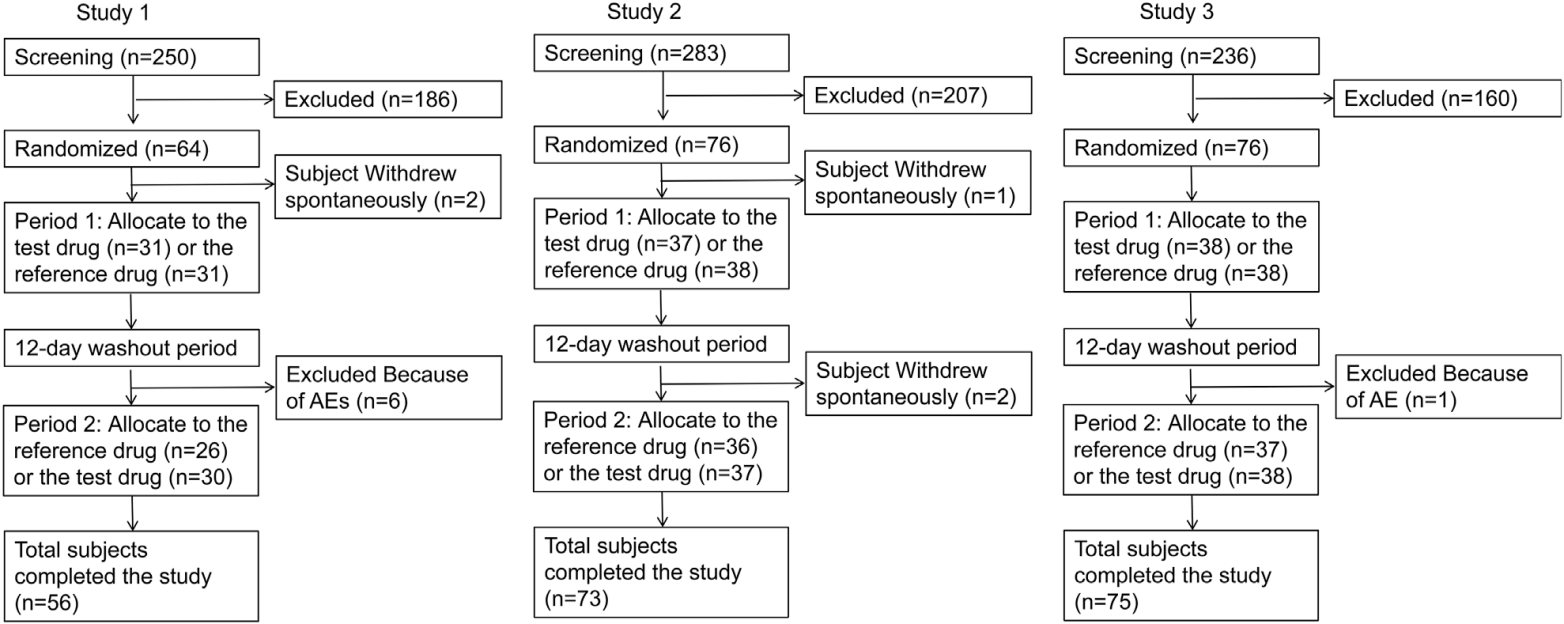
Subjects disposition flow diagram.

Study 3 took place from 2 February 2023, to 13 March 2023, involving seventy-six subjects. One subject withdrew before the second administration due to an AE, resulting in seventy-five subjects completing both treatment periods. Baseline demographics and participant characteristics are detailed in Table 1, while the participant disposition is illustrated in Figure 1.

**TABLE 1:** Demographics and subject characteristics at baseline.

	Study 1	Study 2	Study 3
	n = 64	n = 76	n = 76
Age (years, mean ± SD)	38.31 ± 6.06	38.72 ± 6.11	38.57 ± 6.02
Males (n, [%])	64 (100.00)	76 (100.00)	76 (100.00)
Weight (kg, mean ± SD)	68.49 ± 8.05	69.20 ± 8.45	70.85 ± 7.36
Height (cm, mean ± SD)	169.72 ± 6.99	170.29 ± 5.94	169.44 ± 5.99
BMI (kg/m^2^, mean ± SD)	23.74 ± 1.96	23.81 ± 2.04	24.66 ± 2.00

BMI, body mass index; SD, standard deviation.

### Pharmacokinetics

**FIGURE 2:**
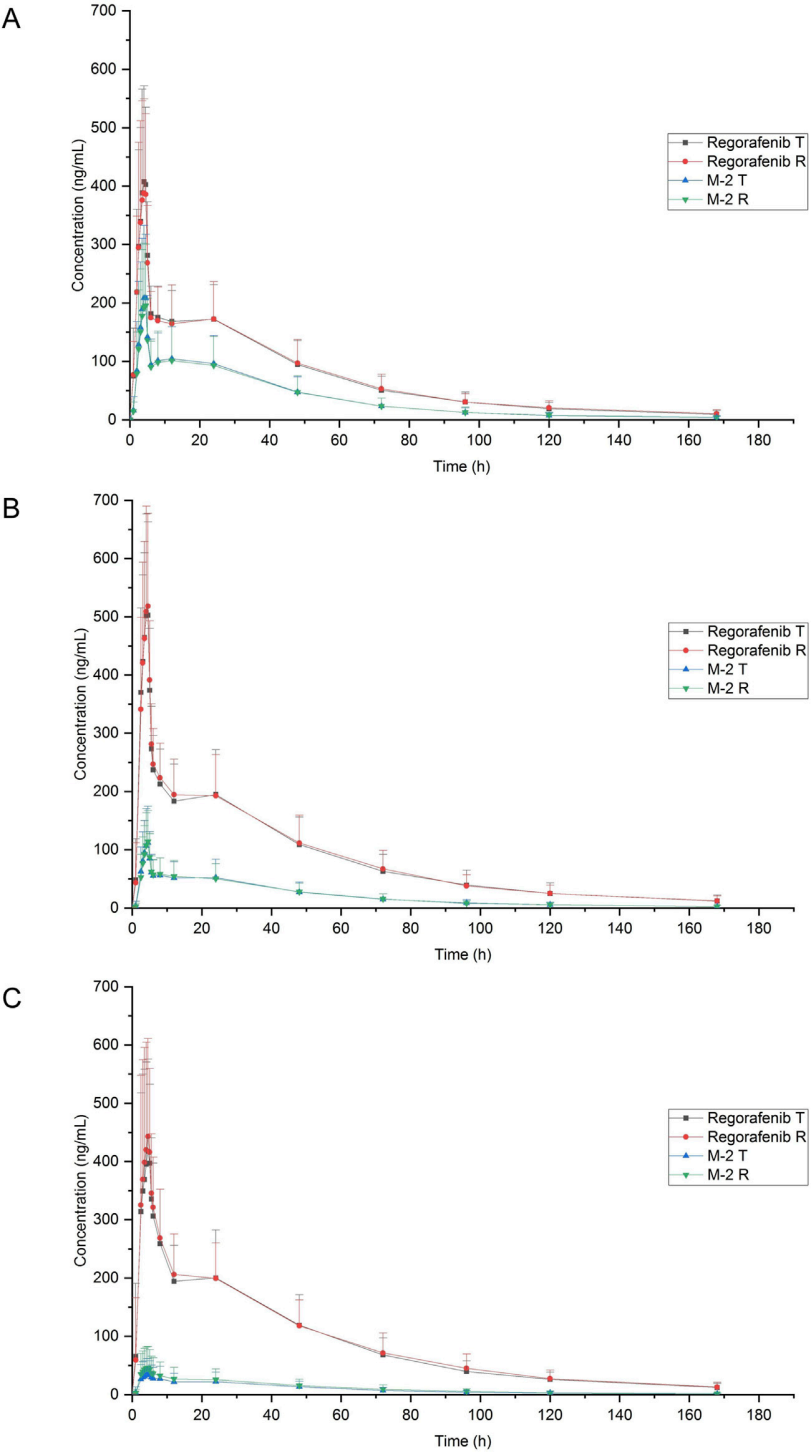
Mean plasma concentration-time profiles of regorafenib and M-2 after a single 40 mg oral dose of reference and test formulation. **(A)** Fasting condition; **(B)** Low-fat condition; **(C)** High-fat condition.

The mean blood concentration-time profiles of regorafenib and M-2 for both the test and reference formulations under three dietary conditions are graphically represented in Figure 2. Summaries of the pharmacokinetic (PK) parameters for both formulations are provided in Table 2. The geometric mean ratios (GMRs) and corresponding 90% confidence intervals (CIs) for the primary PK parameters of regorafenib are presented in Table 3. The GMRs and 90% CIs for C_max_, AUC_0–168h_, and AUC_0–∞_ of the prototype of regorafenib across studies 1, 2, and 3 all fell within the bioequivalence acceptance range of 80.00%–125.00%, thereby establishing bioequivalence. The BE assessment of M-2 for the two formulations was equivalent under fasting and low-fat conditions, but not equivalent under high-fat conditions. However, M-2 data were provided as Supplementary Material only and were not considered as the result of equivalence assessment. Details are presented in Supplementary Table 1.

**TABLE 2:** Pharmacokinetic parameters of regorafenib and M-2 after single oral administration of test and reference regorafenib tablet under different dietary conditions.

Parameter	Study 1 (under fasting conditions)		Study 2 (after low-fat breakfast)		Study 3 (after high-fat breakfast)	
	Test (n = 57)^a^	References (n = 61)^a^	Test (n = 74)^b^	References (n = 74)^b^	Test (n = 75)^c^	References (n = 75)^c^
Regorafenib						
C_max_ (ng/mL)^d^	446.44 ± 160.72	422.79 ± 164.63	527.57 ± 172.33	550.61 ± 159.13	458.04 ± 163.72	490.53 ± 162.15
AUC_0_-_168h_ (ng·h/mL)^d^	11,731.52 ± 3875.79	11,847.75 ± 3899.15	13,851.83 ± 4,731.93	14,072.88 ± 4,501.23	14,499.94 ± 4,875.51	14,952.59 ± 4,513.89
AUC_0_-_∞_ (ng·h/mL)^d^	12,461.02 ± 4,340.35	12,506.63 ± 4,117.17	14,591.99 ± 5,025.91	14,860.75 ± 4,778.35	15,196.66 ± 4,979.98	15,749.82 ± 4,768.15
T_max_ (h)^e^	4.00 (2.5.8.0017)	4.00 (2.24)	4.25 (2.5.5)	4.26 (2.5.5)	4.50 (2.5, 12)	4.50 (2.5, 24)
t_1/2_ (h)^d^	41.49 ± 27.80	38.26 ± 12.85	37.10 ± 10.67	38.74 ± 16.92	36.78 ± 10.67	36.99 ± 12.13
M-2						
C_max_ (ng/mL)^d^	223.15 ± 122.96	205.63 ± 110.32	115.22 ± 65.13	118.44 ± 56.02	39.30 ± 29.06	50.07 ± 38.02
AUC_0_-_168h_ (ng·h/mL)^d^	6,002.27 ± 2,997.18	5,879.63 ± 3151.31	3385.72 ± 1929.00	3369.11 ± 1,630.77	1,507.26 ± 1,042.61	1829.94 ± 1,359.81
AUC_0_-_∞_ (ng·h/mL)^d^	6,222.42 ± 3047.48	6,095.92 ± 3241.20	3535.67 ± 1996.83	3530.94 ± 1,685.83	1,655.85 ± 1,231.13	1960.22 ± 1,389.44
T_max_ (h)^e^	4.50 (2.5.24)	4.50 (2.12)	4.50 (2.5.12)	4.5 (3.5.8)	4.75 (2.5, 48)	4.50 (3, 24)
t_1/2_ (h)^d^	38.49 ± 20.98	34.35 ± 6.69	35.00 ± 9.50	39.65 ± 17.12	43.73 ± 37.26	36.69 ± 11.31

AUC, area under the plasma concentration-time curve; AUC_0-∞_, AUC from time 0 to infinity; AUC_0–168h_, AUC from time 0–168h; C_max_, maximum plasma concentration; t_1/2,_ half-life time; T_max_, time to reach C_max_.

^a^ In the TR sequence, one subject withdrew before administration, and five subjects only completed the first period. In the RT sequence, one subject withdrew before administration, and one subject only completed the first period.

^b^ In the TR sequence, one subject withdrew before administration, and one subject only completed the first period. In the RT sequence, one subject withdrew before administration, and one subject only completed the first period.

^c^ In the TR sequence, one subject only completed the first period. In the RT sequence, one subject’s blood concentration of regorafenib reached C_max_ at the first sampling point after the second period of administration, and only the first period could be included.

^d^ Data were presented as the mean ± standard deviation.

^e^ Data were presented as the median (range).

**TABLE 3:** Bioequivalence evaluation for the primary pharmacokinetic parameters of regorafenib under different dietary conditions.

Parameter	GM (test)	GM (reference)	GMR (test/references)	Intra-CV (%)^a^	90% CI (%)
Study 1 (under fasting conditions)					
C_max_ (ng/mL)	415.51	394.75	105.26	28.72	96.39–114.94
AUC_0_-_168h_ (ng·h/mL)	11,087.65	11,083.90	100.03	20.69	93.81–106.67
AUC_0_-_∞_ (ng·h/mL)	11,744.80	11,684.87	100.51	20.76	94.23–107.21
Study 2 (after low-fat breakfast)					
C_max_ (ng/mL)	498.61	525.00	94.97	26.55	88.40–102.04
AUC_0_-_168h_ (ng·h/mL)	13,052.05	13,380.83	97.54	19.86	92.40–102.97
AUC_0_-_∞_ (ng·h/mL)	13,757.44	14,121.75	97.42	19.00	92.50–102.60
Study 3 (after high-fat breakfast)					
C_max_ (ng/mL)	428.61	462.55	92.66	28.49	85.86–100.01
AUC_0_-_168h_ (ng·h/mL)	13,671.76	14,263.89	95.85	22.27	90.26–101.79
AUC_0_-_∞_ (ng·h/mL)	14,360.07	15,022.43	95.59	21.70	90.15–101.36

C_max_, maximum plasma concentration; AUC_0–168h_, AUC, from time 0–168 h; GM, geometric mean; GMR, geometric mean ratio; CV, coefficient of variation; CI, confidence interval.

^a^ The INtra-CV was estimated under the assumption that T and R were consistent.

### Safety

In Study 1, thirty-three out of sixty-four subjects (53.2%) experienced a total of eighty-eight AEs, eighty-four of which were considered possibly related to the test or reference formulations. Fifty-two and thirty-two AEs were likely related to the test and reference formulations, respectively. All AEs were mild (Grade 1), and all participants fully recovered or showed improvement.

In Study 2, fifty-three out of seventy-five subjects (70.7%) experienced one hundred forty-seven AEs, with one hundred thirty-nine considered possibly related to the test or reference formulations. Seventy-four and sixty-five AEs were likely attributable to the test and reference products, respectively.

In Study 3, forty-four out of seventy-six subjects (57.9%) experienced ninety-one AEs, with seventy-one considered possibly related to the test or reference formulations. Thirty-seven and thirty-four AEs were likely associated with the test and reference products, respectively. Most AEs were asymptomatic abnormalities in laboratory results or vital signs, and no vomiting was reported. No other AEs were likely to have impacted the PK parameters. No hand-foot skin reactions were observed, though one subject experienced dry skin. Most AEs were Grade 1, and all participants fully recovered or improved.

No serious AEs were reported in any of the studies, and there was no statistically significant difference in the incidence of AEs between the test and reference formulations. Further details are available in Supplementary Table S2. The single 40 mg dose of regorafenib was generally safe and well tolerated.

## Discussion

This research conducted a series of PK and bioequivalence (BE) study of a single 40 mg dose of regorafenib in Chinese healthy subjects under difference dietary conditions. The PK studies of the original drug [Stivarga^®^ (regorafenib) label. 2012; Stivarga^®^ summary. 2013; Mross et al., 2012; Strumberg et al., 2012; Sunakawa et al., 2014; Gerisch et al., 2018] were mostly conducted with a dose of 160 mg, therefore, both C_max_ and AUC in our studies (C_max_:394.75 ng/mL; AUC:11,684.87 ng h/mL) were much lower than those at the 160 mg dose level (C_max_:1.25 mg/L; AUC:45.4 mg h/L), which were about 1/4 of that in the study with a 160 mg dosage. However, the T_max_ of this study was consistent with that of the original drug, being approximately 4 h under fasted condition. Meanwhile, with an increase in the fat content of meals, the time to peak concentration was gradually delayed (from 4 h to 4.5 h). Food intake may slightly delay the absorption of regorafenib.

There have been BE studies of regorafenib in the Chinese population before, such as Zhang’s study (Zhang et al., 2021), which had a different design from this research by administering a single oral dose of 160 mg regorafenib to healthy subjects. Considering the safety of healthy subjects, this study set the dose at 40 mg, which also complies with regulatory requirements. Another study (Wang et al., 2024) had a smaller sample size and did not evaluate bioequivalence under low-fat meal conditions. This research, however, took into account the necessity of having a sufficiently large sample size and potential differences under fasting, low-fat, and high-fat fed conditions, resulting in a more comprehensive trial design. Overall, under the three fed conditions with a 40 mg dose, this research successfully analyzed the pharmacokinetic characteristics of Chinese healthy subjects after administration of the original and generic versions of regorafenib, confirming the bioequivalence of the two formulations, thus supporting the marketing application for the generic version.

This food-effect research evaluated the PK of regorafenib and its metabolite M-2 under fasting, low-fat meal, and high-fat meal conditions. In previous food-effect studies of the original drug (Stivarga^®^ (regorafenib) label. 2012; Stivarga^®^ summary. 2013), a high-fat meal increased the mean AUC of regorafenib by 48%, while decreasing the mean AUC of the M-2 and M-5 metabolites by 20% and 51%, respectively. Conversely, a low-fat meal increased the mean AUC of regorafenib, M-2, and M-5 by 36%, 40%, and 23%, respectively, compared to fasting conditions. That result indicated that the low-fat meal state led to the most pronounced changes in the PK parameters of regorafenib and its M-2 metabolite, suggesting a more significant pharmacological effect. Consequently, the original drug label recommends administration after a low-fat meal. However, this does not explain why the effects of high-fat and low-fat diets on M-2 are opposite. Our studies found that as the fat content increased, the AUC of the prototype drug regorafenib gradually increased while the AUC of M-2 gradually decreased, which well explained the impact of fat on the metabolic process. Our findings differ from previous studies, possibly due to two reasons: first, these three studies had a larger sample size; second, the study population was primarily Han Chinese, who may have more consistent hepatic enzyme characteristics. Overall, since regorafenib and M-2 possess similar pharmacological activities, despite the increase in AUC of regorafenib with the rise of fat content in meals, the AUC of M-2 correspondingly decreased. Therefore, the fat content of meals had a relatively small impact on the final drug exposure. These findings provide valuable insights into the clinical application of regorafenib in Chinese patients. Foods with different fat contents have a minor effect on the drug exposure. Thus, it is appropriate to follow the instructions of Stivarga^®^. But there is no need to worry too much about the proportion of fat in the diet.

The safety profile of a single 40 mg oral dose of regorafenib was acceptable, with no significant differences noted between the test and reference formulations. No cases of hand-foot skin reaction were observed, potentially due to the lower dose and extended interval between administrations. Nevertheless, attention to this adverse reaction remains warranted in clinical practice.

This study has some limitations. Based on preclinical data indicating risks to the female reproductive system, the study was exclusively conducted in male participants, and thus, no data are available for female participants.

## Conclusion

The bioequivalence of 40 mg doses of the test and reference formulations of regorafenib was established under fasting and fed conditions (low-fat and high-fat) based on C_max_, AUC_0–168h_, and AUC_0–∞_ parameters. Food intake may slightly increase the exposure of the prototype of regorafenib and delay its absorption. A single 40 mg dose of regorafenib was well tolerated, and no serious adverse events were observed in healthy Chinese participants.

## Data Availability

The original contributions presented in the study are included in the article/Supplementary Material, further inquiries can be directed to the corresponding author.
