# Supplementary material for: A randomized, open-label, two-period crossover study to evaluate the bioequivalence and food effect between two formulations of regorafenib in healthy adult participants
Source: Front Pharmacol. 2025 Mar 19;16:1511558. doi: 10.3389/fphar.2025.1511558 (PMC11962789; doi:10.3389/fphar.2025.1511558)
Supplement: Supplementary file 1 [file Table1.DOCX]

**Supplementary Table** **S1** **Bioequivalence Evaluation for the Pharmacokinetic Parameters of M-2 under Different Dietary Conditions**

| **Parameter** | **GM (Test)** | **GM (Reference)** | **GMR (Test/ Reference)** | **90% CI (%)** |
| --- | --- | --- | --- | --- |
| Study 1 (under fasting conditions) | | | | |
| C_max_ (ng/ml) | 183.71 | 178.23 | 103.07 | 87.65-121.20 |
| AUC_0_-_168h_ (ng·h/ml) | 5132.99 | 5096.61 | 100.71 | 87.86-115.45 |
| AUC_0_-_∞_ (ng·h/ml) | 5364.62 | 5292.01 | 101.37 | 88.85-115.66 |
| Study 2 (after low-fat breakfast) | | | | |
| C_max_ (ng/ml) | 93.77 | 99.30 | 94.43 | 82.01-108.73 |
| AUC_0_-_168h_ (ng·h/ml) | 2805.00 | 2867.69 | 97.81 | 86.50-110.61 |
| AUC_0_-_∞_ (ng·h/ml) | 2946.71 | 3052.85 | 96.52 | 86.07-108.24 |
| Study 3 (after high-fat breakfast) | | | | |
| C_max_ (ng/ml) | 28.40 | 33.15 | 85.68 | 70.76-103.76 |
| AUC_0_-_168h_ (ng·h/ml) | 1131.18 | 1271.24 | 88.98 | 75.23-105.25 |
| AUC_0_-_∞_ (ng·h/ml) | 1285.77 | 1434.20 | 89.65 | 76.67-104.84 |

C_max_, maximum plasma concentration; AUC_0-168h_, AUC from time 0 to 168h; GM Geometric Mean; GMR geometric mean ratio; CV coefficient of variation; CI confidence interval.

**Supplementary Table** **S2** **Summary of Drug-related Adverse Events (AEs) in the Safety Population of Subjects**

|  | **Study 1** | | **Study 2** | | | **Study 3** | |
| --- | --- | --- | --- | --- | --- | --- | --- |
|  | Test (%)  (N=61) | Reference (%)  (N=57) | Test (%)  (N=74) | Reference (%)  (N=74) | Test (%)  (N=76) | | Reference (%)  (N=75) |
| Cardiac disorders | | | | | | | |
| Sinus bradycardia | 9(14.8) | 7(12.3) | 17(23.0) | 15(20.3) | 0(0) | | 0(0) |
| Atrioventricular block first degree | 0(0) | 0(0) | 0(0) | 1(1.4) | 0(0) | | 0(0) |
| Supraventricular premature beats | 0(0) | 0(0) | 1(1.4) | 0(0) | 0(0) | | 0(0) |
| Eye disorders | | | | | | | |
| Conjunctival hyperemia | 0(0) | 0(0) | 0(0) | 1(1.4) | 0(0) | | 0(0) |
| Gastrointestinal disorders | | | | | | | |
| Diarrhea | 0(0) | 0(0) | 1(1.4) | 0(0) | 0(0) | | 0(0) |
| Abdominal distention | 0(0) | 0(0) | 0(0) | 0(0) | 2(2.6) | | 1(1.3) |
| Toothache | 0(0) | 0(0) | 0(0) | 0(0) | 1(1.3) | | 0(0) |
| Mucositis oral | 0(0) | 0(0) | 0(0) | 0(0) | 0(0) | | 1(1.3) |
| Dry mouth | 1(1.6) | 0(0) | 0(0) | 0(0) | 0(0) | | 0(0) |
| General disorders and administration site conditions | | | | | | | |
| Fatigue | 0(0) | 0(0) | 1(1.4) | 0(0) | 0(0) | | 0(0) |
| Hepatobiliary disorders | 0(0) | 0(0) | 0(0) | 0(0) | 0(0) | | 0(0) |
| Cholecystitis | 0(0) | 0(0) | 0(0) | 0(0) | 1(1.3) | | 0(0) |
| Infections and infestations | | | | | | | |
| Upper respiratory infection | 1(1.6) | 1(1.8) | 0(0) | 1(1.4) | 3(3.9) | | 4(5.3) |
| Investigations | | | | | | | |
| Alanine aminotransferase increased | 3(4.9) | 0(0) | 4(5.4) | 3(4.1) | 2(2.6) | | 5(6.7) |
| Aspartate aminotransferase increased | 1(1.6) | 1(1.8) | 2(2.7) | 0(0) | 0(0) | | 4(5.3) |
| Serum amylase increased | 2(3.3) | 0(0) | 0(0) | 0(0) | 0(0) | | 0(0) |
| Serum urea increased | 1(1.6) | 0(0) | 0(0) | 0(0) | 0(0) | | 0(0) |
| Blood bilirubin increased | 0(0) | 0(0) | 0(0) | 1(1.4) | 0(0) | | 0(0) |
| Blood conjugated bilirubin increased | 0(0) | 1(1.8) | 1(1.4) | 2(2.7) | 0(0) | | 0(0) |
| Cholesterol high | 0(0) | 0(0) | 1(1.4) | 0(0) | 0(0) | | 0(0) |
| D-dimer increased | 1(1.6) | 0(0) | 1(1.4) | 0(0) | 2(2.6) | | 0(0) |
| Fibrinogen increased | 0(0) | 0(0) | 0(0) | 0(0) | 0(0) | | 2(2.7) |
| White blood cell increased | 0(0) | 0(0) | 0(0) | 0(0) | 0(0) | | 1(1.3) |
| Neutrophil count increased | 1(1.6) | 1(1.8) | 0(0) | 0(0) | 0(0) | | 2(2.7) |
| Lymphocyte count increased | 0(0) | 1(1.8) | 0(0) | 0(0) | 1(1.3) | | 0(0) |
| Monocyte count increased | 0(0) | 1(1.8) | 0(0) | 0(0) | 4(5.2) | | 4(5.3) |
| Platelet count increased | 0(0) | 0(0) | 0(0) | 0(0) | 1(1.3) | | 0(0) |
| Basophil count increased | 3(4.9) | 1(1.8) | 1(1.4) | 0(0) | 2(2.6) | | 0(0) |
| Neutrophil count decreased | 0(0) | 0(0) | 0(0) | 0(0) | 1(1.3) | | 1(1.3) |
| Lymphocyte count decreased | 0(0) | 0(0) | 0(0) | 0(0) | 3(3.9) | | 1(1.3) |
| Slow heart rate | 8(13.1) | 4(7.0) | 20(27.0) | 11(14.9) | 0(0) | | 0(0) |
| Rapid heart rate | 1(1.6) | 0(0) | 0(0) | 0(0) | 0(0) | | 0(0) |
| QT interval prolongation of ECG | 5(8.2) | 3(5.3) | 10(13.5) | 7(9.5) | 0(0) | | 0(0) |
| Short PR interval of ECG | 1(1.6) | 1(1.8) | 1(1.4) | 1(1.4) | 0(0) | | 0(0) |
| QRS complex prolongation of ECG | 0(0) | 1(1.8) | 2(2.7) | 1(1.4) | 0(0) | | 0(0) |
| ST-segment depression of ECG | 0(0) | 0(0) | 1(1.4) | 0(0) | 0(0) | | 0(0) |
| Elevated blood pressure | 2(3.3) | 2(3.5) | 1(1.4) | 0(0) | 2(2.6) | | 1(1.3) |
| Elevated systolic blood pressure | 1(1.6) | 1(1.8) | 1(1.4) | 0(0) | 1(1.3) | | 0(0) |
| Elevated diastolic blood pressure | 3(4.9) | 1(1.8) | 1(1.4) | 3(4.1) | 1(1.3) | | 1(1.3) |
| Elevated urinary leukocyte | 1(1.6) | 0(0) | 0(0) | 0(0) | 0(0) | | 0(0) |
| Elevated urinary erythrocyte | 0(0) | 1(1.8) | 0(0) | 1(1.4) | 0(0) | | 0(0) |
| Positive urine occult blood | 1(1.6) | 1(1.8) | 0(0) | 4(5.4) | 1(1.3) | | 1(1.3) |
| Positive urine protein | 0(0) | 1(1.8) | 0(0) | 0(0) | 3(3.9) | | 2(2.7) |
| Elevated urinary bilirubin | 0(0) | 0(0) | 0(0) | 0(0) | 1(1.3) | | 0(0) |
| Elevated urobilinogen | 0(0) | 1(1.8) | 0(0) | 0(0) | 0(0) | | 0(0) |
| Positive urine ketone | 0(0) | 0(0) | 0(0) | 1(1.4) | 0(0) | | 0(0) |
| Urinary cast detection | 1(1.6) | 0(0) | 0(0) | 0(0) | 0(0) | | 0(0) |
| Abnormal urine pH | 0(0) | 1(1.8) | 0(0) | 0(0) | 0(0) | | 0(0) |
| Metabolism and nutrition disorders | | | | | | | |
| Hyperglycemia | 1(1.6) | 0(0) | 0(0) | 0(0) | 0(0) | | 0(0) |
| Hyperuricemia | 0(0) | 0(0) | 2(2.7) | 5(6.8) | 0(0) | | 1(1.3) |
| Hypokalemia | 0(0) | 0(0) | 3(4.1) | 0(0) | 1(1.3) | | 0(0) |
| Hypophosphatemia | 0(0) | 0(0) | 0(0) | 1(1.4) | 0(0) | | 0(0) |
| Nervous system disorders | | | | | | | |
| Dizziness | 0(0) | 0(0) | 1(1.4) | 0(0) | 0(0) | | 0(0) |
| Renal and urinary disorders | | | | | | | |
| Urinary frequency | 0(0) | 0(0) | 0(0) | 0(0) | 1(1.3) | | 1(1.3) |
| Respiratory, thoracic and mediastinal disorders | | | | | | | |
| Oropharyngeal pain | 1(1.6) | 0(0) | 0(0) | 0(0) | 1(1.3) | | 0(0) |
| Oropharyngeal discomfort | 1(1.6) | 0(0) | 0(0) | 0(0) | 1(1.3) | | 0(0) |
| Rhinodynia | 0(0) | 0(0) | 0(0) | 0(0) | 1(1.3) | | 0(0) |
